# Antiretroviral Treatment for HIV Elite Controllers?

**DOI:** 10.20411/pai.v5i1.364

**Published:** 2020-05-19

**Authors:** Ezequiel Ruiz-Mateos, Eva Poveda, Michael M. Lederman

**Affiliations:** 1 Clinic Unit of Infectious Diseases; Microbiology and Preventive Medicine; Institute of Biomedicine of Seville; Virgen del Rocío University Hospital/CSIC/University of Seville, Spain; 2 Group of Virology and Pathogenesis; Galicia Sur Health Research Institute (IIS Galicia Sur)-Complexo Hospitalario Universitario de Vigo; SERGAS-UVigo; Vigo, Spain; 3 Division of Infectious Diseases; Center for AIDS Research; Case Western Reserve University and University Hospitals; Cleveland Medical Center; Cleveland, Ohio

**Keywords:** Elite Control, HIV, Immune Activation, Inflammation, Cardiovascular Disease, Transmission

## Abstract

In most HIV-infected persons, the natural history of untreated infection is one of sustained viremia, progressive CD4 T cell depletion with resultant morbidity and mortality. The advent of effective combination antiretroviral therapy (ART) that controls HIV replication has altered this landscape dramatically. Yet a rare population of HIV-infected persons—elite controllers (EC)—can control HIV replication such that plasma levels of virus are “undetectable” without ART. The EC phenotype is heterogeneous, with some subjects durably controlling the virus—persistent elite controllers—and some eventually losing viral control—transient elite controllers. Overall, EC tend to have robust HIV-specific T cell responses and in some cases, mainly in transient elite controllers, elevated activation and inflammation indices that diminish with ART suggesting that endogenous defenses against this persistent pathogen come at the cost of heightened activation/inflammation. A limited data set suggests that cardiovascular disease risk as well as the occur-rence of other morbid events may be greater in the overall EC population than in treated HIV infection. ART in EC decreases activation indices but does not appear to increase circulating CD4 T cell numbers nor do we know if it alters clinical outcomes. Thus, it is difficult to recommend or discourage a decision to start ART in the EC population but the authors lean toward treatment particularly in those EC whose activation indices are high and those who are progressively losing circulating CD4 T cell numbers. Biomarkers that can reliably predict loss of virologic control and immune failure are needed.

## INTRODUCTION

### Definition of Elite Control

Elite controllers (EC) represent a rare subset of persons living with HIV infection who somehow manage to control HIV replication in the absence of antiretroviral therapy (ART). Typically comprising between 0.5% and 1.5% of populations with HIV infection [1–5], in these persons, plasma levels of HIV are below limits of detection by clinical assays. However, EC represent a heterogeneous population with different definitions based on the length of the follow up, viremia detection limits, presence/absence of blips, and/or CD4+ T cell counts [6–8]. Indeed, recent studies were able to identify at least two well-defined phenotypes [9–11]. Overall, persistent EC are those with a length of follow up > 10 years with plasma levels of HIV under the detection limits in the absence of blips and CD4+ T-cell counts > 500 cells/mm^3^; this contrasts with transient EC who with time, lose virologic control (Table 1). The subset of elite controllers characterized as “exceptional elite controllers,” variably described with low levels of HIV antibodies, low HIV reservoir levels including low levels of replication competent virus, low systemic levels of inflammation appear to overlap largely with controllers who durably control HIV replication [9–13].

**Table 1.** Immunologic and virologic characteristics of persistent and transient elite controllers.

	Persistent EC	Transient EC
**Viral Blips** [8, 9, 30]	Absence	Presence
**Immune Activation/Inflammation** [9, 10, 11, 30]	Low	High
**HIV-specific T-cell response levels** [10, 12, 13]	High	Low
**HIV genetic diversity** [10, 13]	Low	High
**HIV Reservoir Size** [9, 10, 12, 13]	Low	Low

In general, EC may represent the far end of a continuum of virologic control as the range of viremia in persons living with HIV infection is broad, yet there are some characteristics of EC that suggest the presence of specific protective mechanisms. Some groups have identified a relatively high prevalence of virologic control among HIV infected persons who began ART relatively early in the course of infection [14]. These “post-treatment controllers” will not be discussed here.

Some instances have been described wherein the viruses of EC have appeared to be defective or less fit [15, 16], however there is increasing evidence that host factors may be particularly important in determining the ability to control HIV replication in EC. Persons with the EC phenotype often carry the HLA-B57 or B27 allele suggesting the importance of adaptive immunity and the role of CD8+ T cells in mediating this protection, however not all EC are HLA-B57+ or B27+ and not all HLA-B57+ or –B27+ persons who are HIV infected are EC. Interestingly, both CD8+ T cell responses tend to be brisk and polyfunctional in persons who control HIV replication [17–19]. Resident CD8+ T cells at lymphoid sites of HIV replication are enriched for expression of genes with effector function [20] and mucosal CD8+ T cells reactive with HIV peptides tend to be more potent and polyfunctional [21]. Moreover, HIV-specific CD4+ T cell responses are characteristically preserved in EC [22] and while it is plausible and likely that these immune defense are important in virologic control, it is also possible that virologic control and the absence of viremia-mediated immune suppression [23] promote a bidirectional environment that also protects T cell function that controls HIV replication.

Intrinsic relative resistance of CD4+ T cells to HIV infection has been found in some [24] studies and both natural killer cells [25] and antibodies, both neutralizing or mediating other activities [26, 27] have been implicated in the control of HIV replication in some EC.

Finally, several of these mechanisms have also served to differentiate persistent and transient elite controllers. Persistent controllers have brisk HIV-specific T-cell responses [10], associated with low viral genetic diversity and variability together with lower measures of the HIV reservoir [9, 10] compared to these indices in transient controllers. Likewise, persistent controllers show lower levels of systemic inflammation [10]. Additionally, these two phenotypes also differ in a peculiar proteomic profile associated with lower levels of inflammation in persistent controllers than in transient controllers [11]. Also metabolomic and lipidomic profiles are distinguishable between these two groups [28]. Additional studies are needed to validate biomarkers that reliably segregate these two subsets of controllers.

### Natural Course of HIV infection in EC

EC tend to have a relatively stable disease course even in the absence of ART with a much slower progression to AIDS defining events and a less frequent CD4 T cell decline to absolute counts < 350/μL [29]. CD4 T cell numbers appear to be stable particularly in EC who do not experience viral blips periods [30]. Yet one cohort study demonstrated frequent hospitalizations among EC and an apparent increased risk for cardiovascular complications in EC when compared to the occurrence of these events in HIV infected persons who were receiving ART [31]. However, analyzing a younger, different cohort with lower prevalence of HCV coinfection, these authors did not find differences in the rate of non AIDS events among EC compared to ART treated persons [32]. Likewise, in the Spanish AIDS Research Network HIV Controllers Cohort (ECRIS), lower incidence of non AIDS events were found in EC than in non controllers [33].

Also among 204 EC from ECRIS, a loss of virologic control occurred at a rate of 4% per year and a 25% or greater decrease in circulating CD4 T cell counts was observed in 6% of subjects [34]. These losses were not always concordant and there were 0.4 AIDS or death events per 100 person-years of follow up in EC [34]. The EC phenotype appeared to be common among HCV co-infected persons in some cohorts [34, 35].

In a number of studies performed among HIV infected persons receiving ART, high levels of inflammatory markers are associated with an increased risk of morbid events [36–38]. Plasma levels of inflammatory mediators including some mediators that were linked to these morbid outcomes were higher in EC than among ART treated subjects [39, 40] but none were linked to levels of residual viremia or CD4 decline [41]. In a small study among 14 EC who lost virologic control (transient controllers) and 18 EC who sustained it (persistent controllers) lower levels of HIV Gag-specific T cell polyfunctionality as well as heightened T cell activation appeared to predict the loss of virologic control [10].

It is not clear whether the heightened inflammation seen in some EC is linked to risk or to protection. In the study by Jacobs *et al*, a large panel of 87 plasma cytokines were examined by multiplexed assays [42]. Of these, 4 were elevated in EC and not in plasmas of non controllers or ART treated persons. When applied in combination, these cytokines could increase expression of the HIV restriction factors interferon inducible transmembrane proteins (IFITM1/2). Whether this effect is operative *in vivo* is not clear as a variety of additional effects on cellular activation and coreceptor expression were observed as well. In a small study performed among 10 EC and in which comparisons were made to subjects reported in earlier studies, there appeared to be elevated plasma levels of monocyte activation markers (sCD163 and sCD14) than seen among ART treated patients and an increased prevalence of coronary artery plaque (by angiography) when compared with healthy donors but not with ART treated patients [43]. In another larger study, EC had greater carotid intima –media thickness (IMT) and higher levels of C reactive protein in plasma than did HIV seronegative controls even when corrected for other risk factors, although there were no differences with ART treated persons [44].

### Treatment of Elite Controllers

Hatano *et al* treated HIV controllers with ART (raltegravir/tenofovir and emtricitibine) for 24 weeks [45]. Using sensitive measurements, they found that ART decreased levels of HIV in plasma and in mucosal sites. Indices of T cell activation fell in peripheral blood and rectal mucosa and there were trends to modest decreases in plasma markers of immune activation (CRP, IL-6, sCD14, and D-dimer) indices that are often linked to morbid outcomes in HIV infection. Circulating CD4+ T cell counts did not increase. It should be noted that only four of these 16 subjects had plasma viral load levels less than 40 copies/mL at treatment initiation. In these four subjects authors found the same results regarding sensitive viral load assays, but no differences were found in T cell activation indices. In another study of 35 controllers of whom 11 were EC, administration of ART regimen for up to 48 weeks, decreased indices of CD4+ and CD8+ T cell activation and decreased plasma levels of IP-10 but had no effect on circulating CD4+ T cell counts and actually increased plasma levels of the monocyte activation marker sCD163. The effects of treatment on activation indices was greater in those persons with higher levels of pre-treatment viremia [46]. In another small study, Kim *et al* treated four EC with a standardized ART regimen (raltegravir/tenofovir/emtricitibine) for 9 months. At baseline, levels of IL-6 and D-dimer were higher and CD4/CD8 ratios were lower in these persons than in uninfected controls. Treatment increased CD4/CD8 ratios but did not affect IL6 levels, D-dimer levels or indices of microbial translocation or gut CD4 T cell subsets [47]. In a larger study of EC where virologic control was defined as plasma levels of virus < 400 copies/mL, EC were treated with ART [48]. In contrast to persons who were not virologic controllers at ART initiation, the treated EC did not experience the typical rapid first phase CD4 T cell increase in blood [49, 50] that is thought to represent a redistribution of sequestered lymphocytes from lymphoid tissues [51, 52], but their second phase slower CD4 T cell increase was similar to that experienced by viremic subjects [48].

In the current treatment era, there is increasing emphasis on the development of strategies to cure HIV infection [53] and in this regard, a reservoir of immune cells latently infected with replication competent HIV represents a barrier to HIV eradication [54]. Chun *et al* demonstrated that the pool of circulating CD4+ T cells that contained replication competent HIV was diminished in EC after ART [55] suggesting that this pool is maintained in EC by ongoing HIV replication and that application of ART in EC can get patients “closer” to cure.

It is important to note these studies did not discriminate between persistent and transient elite controllers and we don't know if ART affects these two subsets of EC differently. The validation of biomarkers distinguishing these two groups of controllers with undetectable viremia would be very useful to gauge the effects of ART in these populations.

## SUMMARY

### Rationale for antiretroviral treatment (or not) for elite controllers

Most EC can maintain control of HIV replication, while some lose control over time; we are not yet able to reliably discriminate between these groups before control is lost. A marker or collection of markers that reliably distinguish between these groups would be helpful in deciding whether antiretroviral treatment is warranted. While confirmatory data from large studies are needed, emerging data provide some distinction between transient and durable controllers. Specifically, the absence of viral blips [9], lower plasma inflammatory chemokine levels [10, 13], lower cellular proviral DNA levels [9, 10, 12], lesser viral diversity [10, 13] and brisk HIV-specific T-cell response [10, 12] and absence of replication-competent virus in blood samples [12, 13] may allow distinction of durable controllers from those who are at risk for losing this status. Recent studies identifying proteomic and metabolomics signatures also may prove useful in distinguishing these groups [11, 28]. Most EC remain well clinically but there is some evidence that EC are at risk for cardiovascular disease and may experience more frequent hospitalization events, although this finding has not been reproduced. Immune activation is increased in some EC and some of these activation indices are linked to risk for morbid events (including cardiovascular events). Emerging evidence suggests that ART can decrease the magnitude of residual HIV replication and decrease some elevated activation indices. It is likely that some component of this activation is a consequence of the sustained activation of immune defenses against HIV that are protective as HIV-specific immune responses diminish when virus replication is attenuated pharmacologically rather than immunologically. Thus it is reasonable to propose that endogenous immune control of HIV may come at a cost, at least in a subset of EC. So should EC be treated with ART? We still don't know whether ART-induced control of HIV replication is associated with demonstrable clinical benefit in this setting and it isn't likely that a clinical trial will be powered to test this question, and although modern ART regimens are relatively well-tolerated, different regimens have recognized toxicities such as weight gain [56, 57], lipid and other metabolic perturbations [58], and bone and renal effects [59]. The balance swings towards recommending ART to EC who lose virologic control or are at greater risk for loss of virologic control and to viremic controllers in whom activation indices fall more dramatically with treatment. It is less clear that ART confers clinical benefit to EC with high CD4+ T-cell counts and low levels of inflammation. Some EC may be comforted with ART initiation hoping that this might provide additional protection against transmission to their sexual partners. Nonetheless to our knowledge there are no documented instances of sexual HIV transmission from an EC and a very small study has failed to detect HIV sequences in the semen of four EC but did detect sequences in persons with measurable virus in plasma [60]. Too, an argument can be made to initiate ART in EC before CD4 T cell numbers fall as these numbers do not appear to recover with ART. In this sense, for those EC with decreased CD4 T cell counts, trials of immunomodulators together with ART should be considered. In summary, we would lean more towards recommending a well-tolerated treatment regimen for EC, particularly those whose activation indices are high and those who progressively lose circulating CD4 T cell numbers but there are insufficient data to support such a recommendation definitively. There is a need to find the most predictive biomarkers that can reliably identify those EC who will lose virologic control and those who will experience immunologic failure. This information could help define a population of EC more likely to benefit from intervention as we learn more about the determinants of virologic control, immune defense, and the characteristics of functional cure or possibly even HIV eradication.
